# Efficacy and safety of continuous passive motion and physical therapy in recovery from knee arthroplasty: a systematic review and meta-analysis

**DOI:** 10.1186/s13018-024-04536-y

**Published:** 2024-01-13

**Authors:** Zhengfeng Jia, Yan Zhang, Wupeng Zhang, Cheng Xu, Wanheng Liu

**Affiliations:** 1 Department of Orthopedics, The Fourth Medical Center of Chinese, PLA General Hospital, Beijing, China; 2 National Clinical Research Center for Orthopedics, Sports Medicine and Rehabilitation, Beijing, China; 3 Graduate School of Medical School of Chinese PLA Hospital, Beijing, China; 4 Department of Quality Management, PLA Rocket Force Characteristic Medical Center, Beijing, China; 5 School of Medicine, Nankai University, Tianjin, China

**Keywords:** Total knee arthroplasty, Continuous passive motion, Physical therapy, Range of motion, Meta-analysis

## Abstract

**Background:**

Continuous passive motion (CPM) is commonly used as a postoperative rehabilitation treatment, along with physical therapy, for postoperative knee rehabilitation. However, the comparison between the two in terms of efficacy in postoperative knee replacement recovery is unclear.

**Purpose:**

To compare efficacy and safety of combined CPM versus physical therapy alone in postoperative rehabilitation after knee arthroplasty.

**Methods:**

PubMed, Embase, and Web of Science databases were used to retrieve and access clinical studies on the efficacy of CPM compared with physical therapy. Review Manager software was used for study publication bias assessment and data analysis based on inclusion criteria.

**Results:**

A total of 6 articles covering 557 patients were included in the study. In terms of range of motion (ROM), passive knee flexion was similar between CPM and physical therapy (PT) (WMD, − 0.17; 95% CI,  − 0.98–0.64; *p* = 0.68). At long-term follow-up, passive knee extension was similar between CPM and physical therapy (PT) (WMD,  − 0.28; 95% CI,  − 1.47 to  − 0.92; *I*^2^ = 65%, *p* =0.65). In addition, CPM generates significantly higher in length of stay (WMD, 0.50; 95% CI,  − 0.31 to 0.69; *I*^2^ = 3%, *p* < 0.001). CPM generates significantly higher treatment costs and incurs more care costs relative to physical therapy.

**Conclusion:**

Compared to PT, combined with CPM failed to significantly improve ROM of the knees and patient’s satisfaction. In addition, CPM treatment significantly increased the cost of hospitalization.

**Supplementary Information:**

The online version contains supplementary material available at 10.1186/s13018-024-04536-y.

## Introduction

Postoperative knee rehabilitation is paramount to maintaining joint motion function [1–4]. The incidence of knee stiffness is reported to be as high as 35% with no or inappropriate rehabilitation [2, 4–6] and significantly affects patient quality of life and satisfaction. Clinically, two intervention strategies can be used to guide patients in postoperative rehabilitation: continuous passive motion (CPM) and traditional physical therapy. CPM was introduced in the 1970s and relies primarily on moving mechanical clips to improve joint mobility and thereby achieve improvement [7–9].

CPM has a positive biological effect on tissue healing, edema, and hematoma [10–12]. Vasileiadis et al. [13] confirmed the role of CPM in the maturation of heterotopic ossification by performing CPM rehabilitation in a 46-year-old male patient with right deviation. Stopping the progression and maintenance of heterotopic ossification became a useful aid in increasing joint mobility. Traditional physical therapy mainly includes dynamic floor exercises, suspension, gait training, closed chain exercises, open chain exercises, and pedal exercises, and the basic idea is an active activity. At present, both rehabilitation strategies are used to guide postoperative rehabilitation, but there is high controversy in the industry regarding the clinical application of both. Therefore, it is extremely important to conduct high-quality clinical evidence-based studies to explore reasonable rehabilitation strategies after knee surgery to guide clinical practice.

Previous studies have reported that the use of CPM has advantages over physical therapy, including reduced swelling, faster return of joint mobility, and reduced analgesia [14]. However, there is still a great deal of controversy about whether it is beneficial for patients' postoperative recovery in the past two decades of research [4, 15–18]. Many researchers support these benefits; on the contrary, many studies show that the advantages of CPM compared to physical therapy are not as clear [1, 2].

Hence, based on previously presented evidence from high-quality randomized controlled trials, our evidence-based study aimed to determine the effectiveness of CPM compared to physical therapy in postoperative orthopedic rehabilitation, comparing key outcomes including knee range of motion (ROM), The Western Ontario and McMaster University Osteoarthritis Index (WOMAC) pain scores, length of stay, satisfaction of patients, postoperative complications, and medical costs.

## Method

This systematic review and meta-analysis following the Preferred Reporting Items for Systematic Reviews and Meta-Analyses (PRISMA) Statement protocol. This study was registered in the International Prospective Register of Systematic Reviews (PROSPERO) (CRD42023410252).

### Search strategy and eligibility criteria

PubMed, Embase, and Web of Science databases were searched. We take PubMed as an example to demonstrate the search strategy for this study (Additional file 1: Appendice 1). We developed specific search strategies for each database, and references of the identified studies were checked for potential eligibility. Relevant clinical outcomes published in January 2000 and April 2023 were retrieved.

Our inclusion criteria for this meta-analysis included: (1) publications comparing the results for both (physiotherapy interventions including active ground exercise, suspension, gait training, closed chain exercise, open chain exercise, and pedal exercise in the control and experimental groups, and CPM in the experimental group); (2) randomized controlled trial and clinical study; (3) the sample size is feasible and the statistical analysis is scientific; (4) primary selection of patients after knee arthroplasty; (5) published literature in English.

Using a standardized data form, we extracted several data elements from the included studies, and two investigators (JZFand WDF), independent of each other, extracted and screened the literature as well as the data according to the inclusion as well as data extraction. If any disagreements arose, they were resolved by discussion or validation by a third-party investigator (XC).

### Data abstraction

We extracted general details and categories mainly including (1) demographics, (2) study characteristics, (3) outcome and prospective measures. Patient statistics included gender, age, and the total number of patients. Characteristics of the trial included author, publication date, study type, CPM, or physical therapy. Outcome measures for this study included ROM (active knee flexion extension and passive knee flexion extension), pain, function, complications, length of hospital stay, and patient satisfaction and were cross-checked.

Prognostic indicators such as postoperative pain were evaluated using the Western Ontario and McMaster University Osteoarthritis Index (WOMAC) score and Visual Analog Scale (VAS) score. The WOMAC Osteoarthritis Index [19] was developed by Bellamy et al. and is one of the most commonly used, patient-reported prognostic indicators for patients with lower extremity osteoarthritis. The WOMAC contains 24 items covering three dimensions: pain (5 items), stiffness (2 items), and function (17 items). The WOMAC has been extensively tested for validity, reliability, feasibility, and responsiveness over time. The VAS has been used since the 1920s to measure intangible indicators of pain, quality of life, and anxiety, and in recent years, the VAS has become a very popular tool for measuring pain [20].

### Risk of bias

The quality of the included studies was assessed independently by two reviewers. In this regard, the Jadad Scale (four categories: (1) Randomization, (2) Concealment, (3) Blinded, and (4) Withdraw or drop-out) for RCT , The Cochrane Risk of Bias (ROB) tool for randomized controlled trials was used to assess the methodological quality of the included studies in our study [13]. Each standard was grouped into three various categories of "low risk", "high risk", or "unclear risk" of bias, and then, the quality of the randomized studies was determined according to institutional health research and quality standards.

### Statistical analysis

We used RevMan version 5.4 Review Manager software for meta-analysis. Weighted mean differences (WMDs) were used to represent the results for continuous data, and 95% CI was used for interval estimation. If *p* < 0.05 was satisfied suggesting that the difference was statistically significant. Meanwhile, the heterogeneity test was performed on the included literature, and when *p* ≥ 0.10 and *I*^2^ ≤ 50%, there was no significant heterogeneity, and the fixed-effect model was used to combine the effect sizes for analysis; if *p* < 0.10 and *I*^2^ > 50%, it indicated that the heterogeneity among the included studies was large, and the sources of heterogeneity were further examined, and after excluding the obvious heterogeneity, the randomized effect model was applied for analysis [21].

If the heterogeneity between studies is significant, subgroup or sensitivity analyses are required to clarify the source of heterogeneity. Trials are subject to clinical and methodological differences, and in this study subgroup analysis based on available data according to follow-up time was performed to generate a final forest plot for description.

## Results

**Fig. 1:**
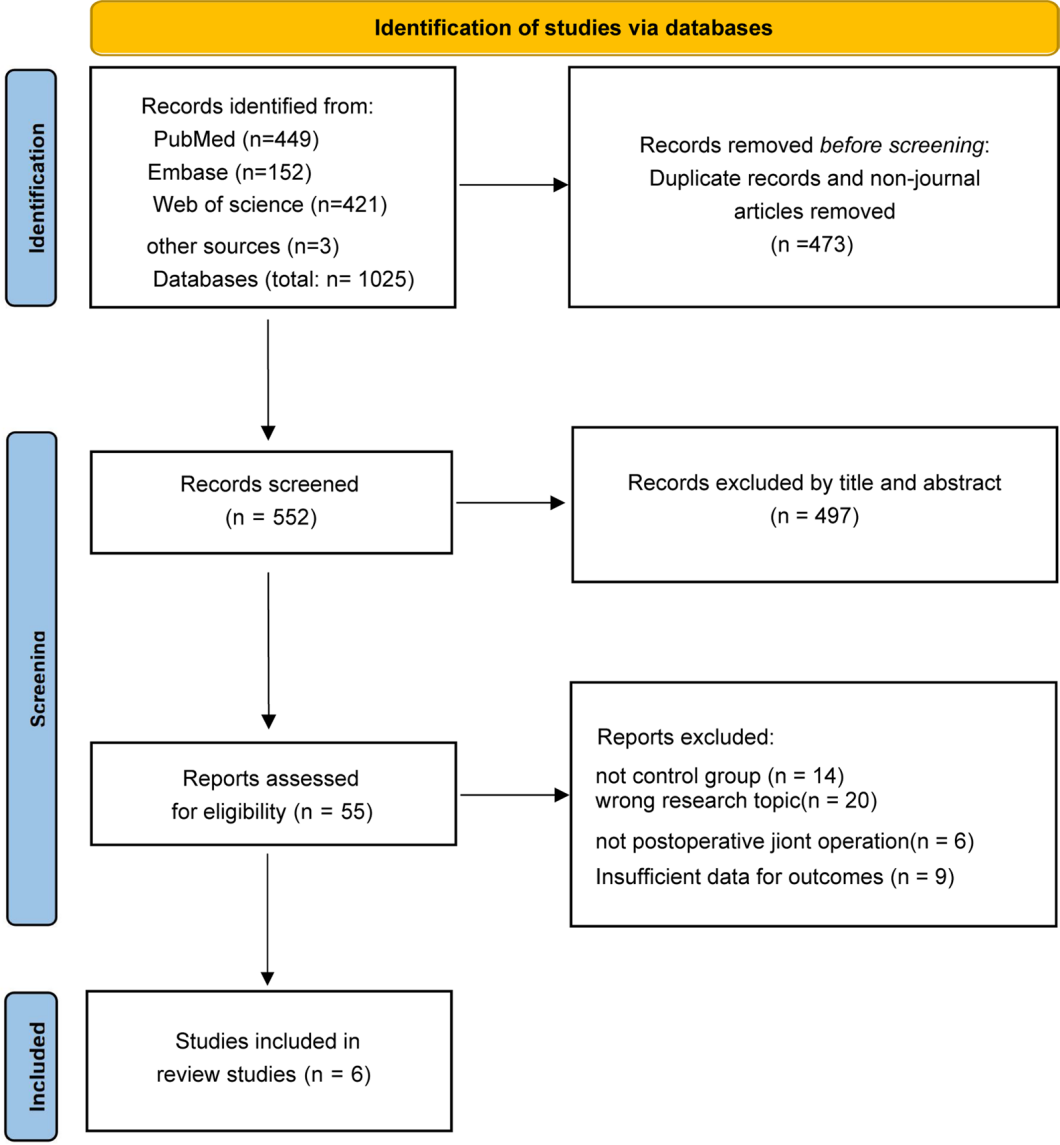
PRISMA flowchart

A total of 1025 publications was retrieved according to the search method, and a total of 6 clinical articles were screened for inclusion in the analysis based on the minimum standard. Figure 1 represents the screening process. Six direct comparisons of 557 cases of CPM after TKA as well as other physiotherapy RCT were included in this meta-analysis [8, 22–26]. The baseline information for these studies is listed in Table 1.

**Table 1:** Basic characteristics of the included papers

Author	Year	Nation	CPM	PT	Age (Mean ± SD)	Sex, Men (%)	Outcomes
Joshi et al. [24]	2015	American	50	50	CPM: 68.5 ± 7.8 PT : 70.5 ± 8.7	CPM: 40% PT : 24%	ROM; Complication; WOMAC; PAQ scores; Discharge location; Cost;
Lenssen et al. [8]	2008	Netherlands	30	30	CPM: 64.1±8.1 PT : 65±9.1	CPM: 40% PT : 30%	ROM-active knee flexion; ROM-passive knee flexion ROM-active knee extension; ROM-passive knee extension Pain, function (WOMAC, Knee Society Score); Pain medication; Satisfaction with treatment; Satisfaction with treatment results; Compliance; Quantity, duration and kind of treatment;
Mau-Moeller et al [26]	2014	Germany	19	19	CPM: 67.1 ±8.8 PT : 68.8 ±8.0	CPM: 63% PT : 53%	passive knee flexion range of motion; active knee flexion range of motion; active and passive knee extension ROM; static postural control; physical activity; pain; length of hospital stay as well as clinical; functional and quality-of-life outcomes (SF-36, HSS and WOMAC scores);
Schulz et al. [25]	2018	Germany	38	38	CPM: 71.0± 8.0 PT : 69.0 ± 8.0	CPM: 44% PT : 52%	Pre-op Flexion; Discharge; Length of stay in days;
Gil‑González et al.[23]	2022	Spain	105	115	CPM: 74.2±6.8 PT : 73.3±6.9	CPM: 36% PT : 39%	ROM-active knee flexion; ROM-passive knee flexion ROM-active knee extension; ROM-passive knee extension Pain medication;
Bruun-olsen et al. [22]	2009	Norway	30	33	CPM: 68.0±10.0 PT : 71.0±10.0	CPM: 27% PT : 33%	Knee circumference; Pain intensity (VAS 0 – 100); Active knee flexion; Passive knee flexion; Active knee extension; Time Up and Go;40 m walking test;

*SF-36* 36-item Short Form Health Survey; *VAS* Visual Analog Scale; *ROM* range of motion; *PAQ* patient-administered questionnaire

### Range of motion

A comparative analysis of passive knee flexion, passive knee extension, active knee flexion, and active knee extension included in the study was mainly conducted to compare the range of knee motion at different periods.

#### Passive knee flexion

**Fig. 2:**
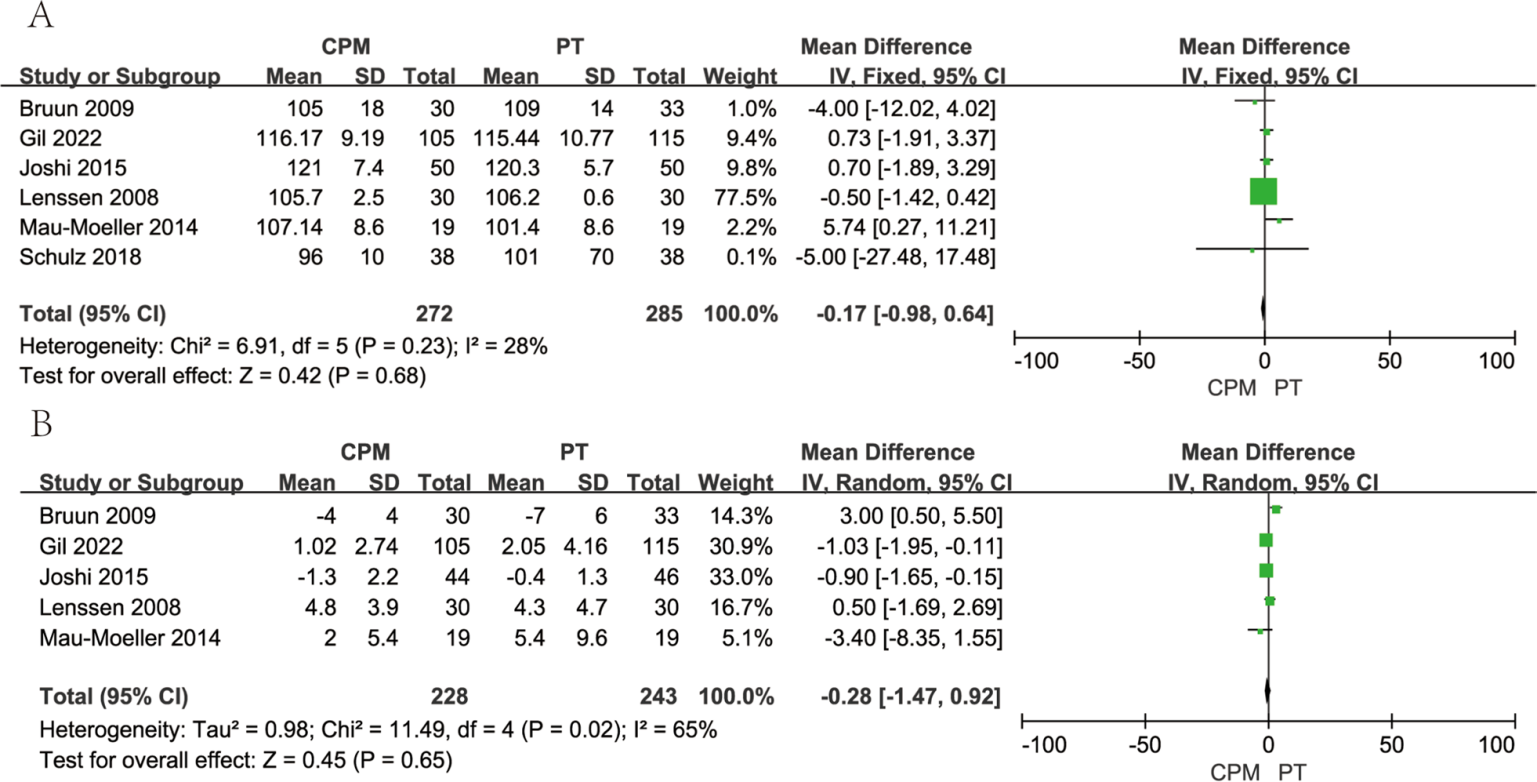
Forest plots of range of motion. **A** Passive knee flexion; **B** passive knee extension

For short-term postoperative recovery, CPM produced better results in the first three days of postoperative recovery compared to physical therapy, and six studies reported long-term (3-month postoperative follow-up) results for passive knee flexion, and we analyzed the results using a random-effects model in which WMD was similar between the experimental and control groups (WMD,  − 0.17; 95% CI,  − 0.98 to 0.64; *p* =0.68). There was no clear evidence of statistically significant heterogeneity throughout the analysis (*I*^2^=28%; *p* = 0.23) (Fig. 2A).

#### Passive knee extension

A total of five studies with 471 patients was analyzed, and we used a random-effects model to analyze the results. There were no significant differences between the two groups at long-term follow-up (WMD,  − 0.28; 95% CI,  − 1.47 to  − 0.92; *I*^2^=65%, *p* = 0.65) (Fig. 2B).

### Length of hospitalization

**Fig. 3:**
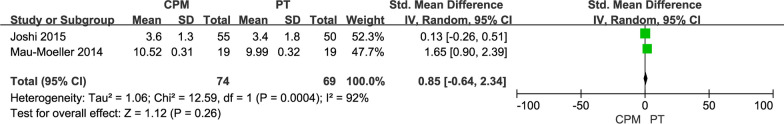
Forest plots of length of hospital

A total of 74 patients were included in 2 studies for analysis of length of stay; CPM generates significantly higher in length of stay (WMD, 0.50; 95% CI,  − 0.31 to 0.69; *I*^2^=3%, *p* < 0.00001) (Fig. 3).

### Pain evaluation

**Fig. 4:**
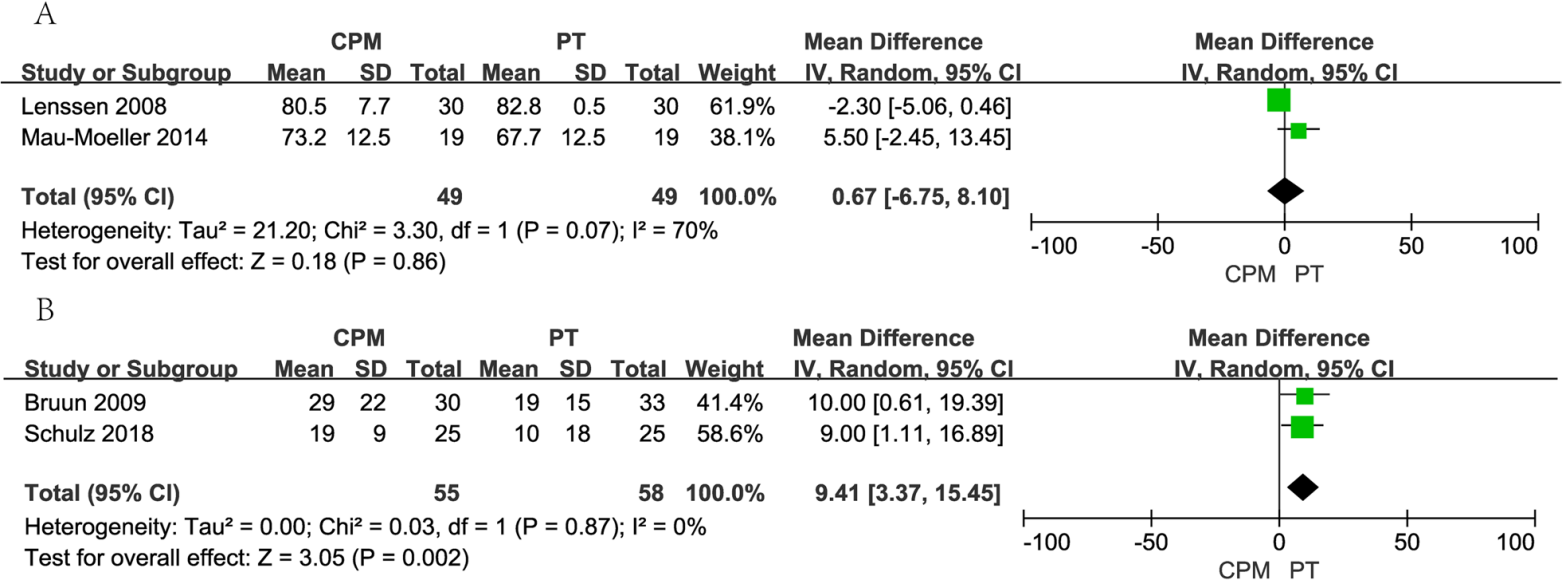
Forest plots of pain scale. **A** WOMAC; **B** VAS

Two studies were scored by WOMAC and analyzed by taking a random-effects model (WMD, 6.75; 95% CI,  − 6.75 to 8.10; *p* = 0.86), with a large heterogeneity between the studies' results. The experimental group scored slightly higher on the WOMAC functional difficulty score, but no significant differences were found after two weeks or on any follow-up measures (Fig. 4A). Moreover, two studies were scored by VAS and analyzed by taking a random-effects model (WMD, 9.41; 95% CI, 3.37–5.45; *p* = 0.002), with a large heterogeneity between the study results. The experimental group scored slightly higher on the VAS functional difficulty score. The VAS was performed, and there was a significant difference between the experimental and control groups (Fig. 4B).

### Satisfaction with treatment

For most patients, their status (perceived outcomes) was "better" compared to preoperative. Patients were generally satisfied with their treatment and outcomes in both the experimental and control groups. The CPM group also did not show a significant advantage in terms of patient-perceived outcomes [24].

### Cost in hospital

Compared with other physical treatments [24], CPM generates significantly higher treatment costs and incurs more care costs.

### Risks of bias

**Fig. 5:**
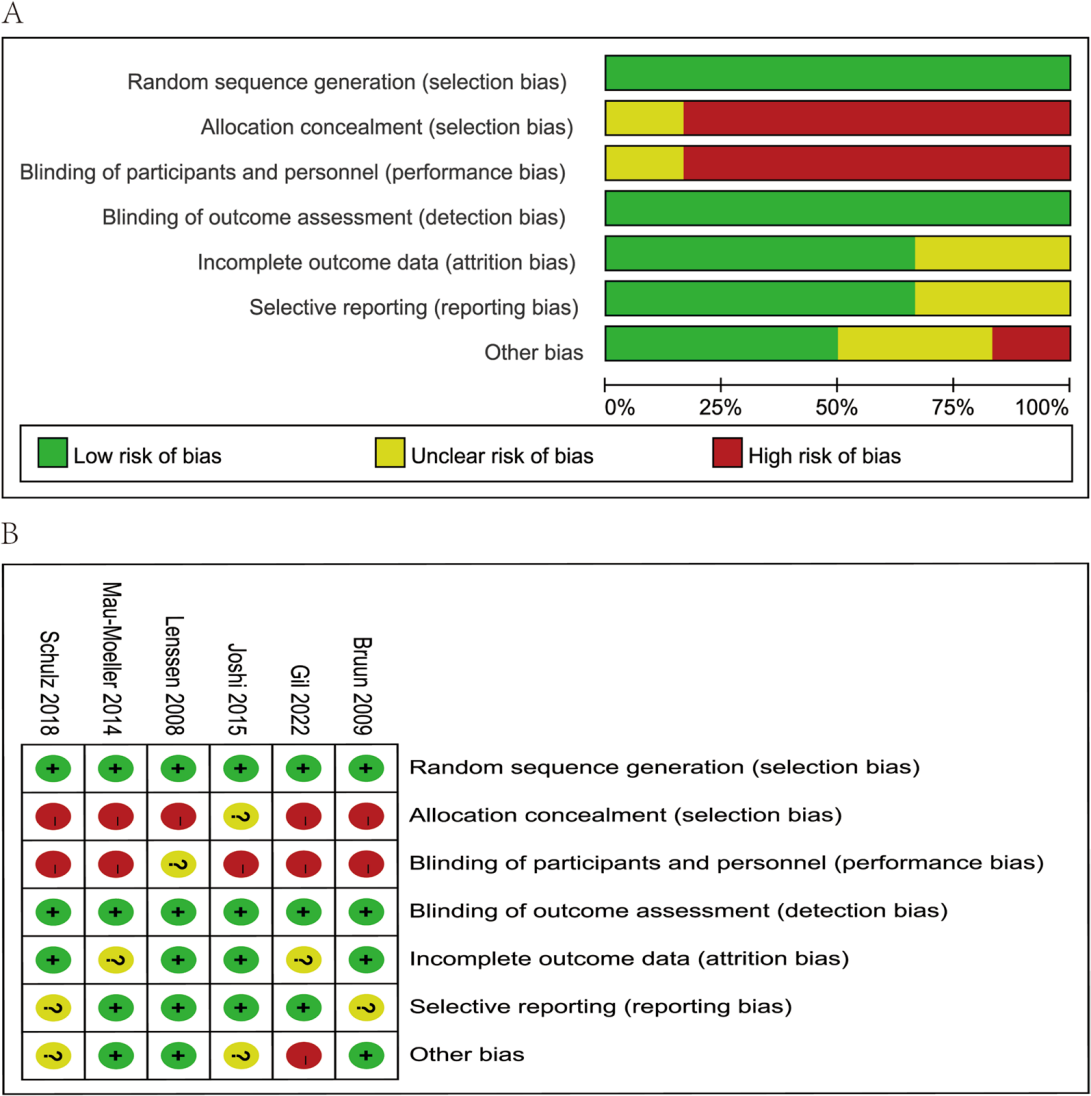
**A** Assessment of the risk of bias. The traffic lights with “x,” “+,” and “−” represent that the corresponding domains are of high, low, and unclear risk of biases, respectively; **B** risk of bias summary. The plus sign means low risk, the question mark means unclear risk, and the minus sign means high risk.

All six included RCTs were unblinded. The six RCTs were relatively well designed with a Jadad score range from 4 to 6 points, which indicated that they were of high quality. The Jadad score is summarized in Table 2. None of the included literature mentioned allocation concealment; the methodological assessment of the quality of the included literature is shown in Fig. 5A, as CPM requires patient consent and signed informed consent, so such studies were unblinded and highly biased in the blinded method. Of the 7 risks of bias domains (blinding of participants and personnel, performance bias) proved to have a high risk of bias. The graph shows "+" for attainment and "-" for non-attainment. Figure 5B shows the quality assessment of each entry of the methodological assessment.

**Table 2:** Quality assessment by the Jadad scale for RCT

Authors	Randomization	Concealment	Blinded	Withdraw or drop-out	Total
Joshi et al. [24]	2	2	1	1	6
Lenssen et al. [8]	1	1	1	1	4
Mau-Moeller et al. [26]	1	2	1	1	5
Schulz et al. [25]	1	1	1	1	4
Gil‑González et al. [23]	1	1	1	1	4
Bruun-olsen et al. [22]	1	2	1	1	5

*RCT* randomized control trial

## Discussion

The present study finds that combined with CPM did not significantly improve postoperative functional recovery compared to physical therapy. There was no difference between the two in terms of time to discharge and patient satisfaction. Overall, CPM did not show an advantage in postoperative patient recovery. Rather, it was associated with increased equipment costs and costs of care. Therefore, the current findings are insufficient to support the routine use of CPM to facilitate the recovery process after arthroplasty. In addition, the heterogeneity of included studies was significant. However, we performed a subgroup analysis of WMD to investigate the source of heterogeneity. The association between CPM and ROM is described at different times, i.e., baseline, day 3 or when the maximum value is reached, probably because these times are highly dependent on the time and angle set by the CPM device. Nonetheless, our subgroup analysis showed that regardless of when ROM was measured, the increase in CPM still produced the same results as physical rehabilitation.

Although several previous studies have confirmed that CPM improves ROM only in the initial postoperative period and does not have much effect on long-term postoperative recovery, this is consistent with the results of our present meta-analysis. The association between CPM and ROM was described at different times, i.e., baseline, day 3, or at the time of maximal value, possibly because these times were highly dependent on the timing and angle of the CPM device settings. However, our subgroup analyses showed that regardless of when ROM was measured, an increase in CPM still produced the same results as physical recovery. Yang et al [27] found that CPM use was not frequently associated with improved knee ROM and functional outcomes from hospital discharge to a final follow-up. In our study, the analysis of patient satisfaction was added, as well as the conclusion that CPM generates more inpatient spending and longer hospital stays. In actual clinical practice, however, the use of CPM devices remains the standard of care in many institutions for rehabilitation [28], although the provision of CPM to patients has now been shown to be associated with insignificant long-term benefits and the short-term therapeutic role of the procedure remains controversial [16, 29]. The primary goal of using a CPM device is to increase short-term postoperative knee ROM, as several studies have reported short-term efficacy of CPM in improving CPM [30], Although most studies have shown nonsignificant results for CPM, CPM is also heavily used, which is related to subjective patient factors as well as recovery expectations, and should be validated by including a larger sample of patients for follow-up. Lee et al studied new CPM machines compared to previous conventional CPM machines to form a clinical assessment of the usefulness and effectiveness of seated CPM machines in patients undergoing total knee arthroplasty, using more objective tools such as digital inclinometers and handheld dynamometers to measure ROM [29].

We clarified that the difference in the effect of CPM and PT on patients' motor function recovery was not significant. On this basis, the patient's satisfaction is important [14]. Several previous studies have shown no statistical difference between the two in terms of patient satisfaction. In Gatewood et al. [31] by analyzing the efficacy of the means of rehabilitation after knee surgery, it was noted that CPM did not improve in terms of patient satisfaction. Wirries et al. [32] prospectively randomized the analysis of patient satisfaction with CPM after TKA through 40 patients, using the WOMAC and the Knee Social Score (KSS), to assess patient satisfaction and knee function, ultimately concluding that there was no significant difference between the both. Our findings also show that CPM does not improve patient satisfaction, possibly because CPM does not show benefit in any of the outcome indicators assessed, provides additional costs, and requires additional training for implementation [33].

In the study conducted by Joshi et al [32], two patients in the CPM group had postoperative complications. One patient was discharged with an acute quadriceps tendon tear and the other had a deep hematoma. One patient in the no-CPM group had a very deep wound dehiscence after a fall. Mau-Moeller et al. [26] systematically evaluated the effectiveness of TKA's new active sling inpatient ROM exercise program; this physical therapy was easy to perform during hospitalization and was less expensive than CPM treatment. Musa Eymir et al.  [34] held that AHSE (active heel gliding exercise) therapy provides more practical rehabilitation and leads to beneficial outcomes for patients with TKA. Therefore, their active exercise approach that encourages patients to participate in rehabilitation should be the first choice for acute postoperative rehabilitation after TKA rather than CPM.

Postoperative knee rehabilitation is essential to maintain joint motor function and significantly affects the quality of life and satisfaction of patients. This study describes in detail the clinical applicability of CPM and PT through meta-analysis, which is of great significance for the selection of rehabilitation exercises and the development of the next rehabilitation program for patients in clinical practice. Meanwhile, our meta-analysis also has some limitations. Firstly, CPM protocols and follow-up periods were inconsistent across all studies, which may lead to the possibility of bias. The long-term impact of CPM should be further assessed. Furthermore, due to the nature of the CPM equipment, it was not possible to blind the subjects to CPM grouping. In addition, some patients had received TKA before this study and therefore knew that the use of CPM devices as standard, could lead to effects that could have uncontrolled patient implications. Therefore, an assessment of the risk of bias revealed a generally high risk of bias in allocation concealment (selection bias) and participant blindness (performance bias). In the case of CPM application, however, these situations are unavoidable. These inconsistent results may be due to inappropriate matching of the CPM machine to the patient as well as measurement errors in ROM between studies.

## Conclusion

Combined with CPM did not significantly improve postoperative functional recovery relative to physical therapy. There was no difference between the two in terms of time to hospital discharge and patient satisfaction. Overall, CPM did not show superior benefits for postoperative patient recovery. On the contrary, it was associated with increased equipment costs and care expenses. Therefore, the results of the current study are insufficient to support the routine use of CPM to facilitate the recovery process after arthroplasty. We believe that as CPM is used more in orthopedics, further optimization of measurement structures and device innovations are needed for additional evaluation.

### Supplementary Information

**Additional file 1:** Appendice 1.

## Data Availability

Relevant data can be available by contacting the corresponding author.

## Abbreviations

CPM: continuous passive motion
PT: physical therapy
ROM: range of motion
