# Supplementary material for: Efficacy and safety of continuous passive motion and physical therapy in recovery from knee arthroplasty: a systematic review and meta-analysis
Source: J Orthop Surg Res. 2024 Jan 13;19:68. doi: 10.1186/s13018-024-04536-y (PMC10787984; doi:10.1186/s13018-024-04536-y)
Supplement: Supplementary file 1 — Additional file 1: Appendice 1. [file 13018_2024_4536_MOESM1_ESM.docx]

**Appendice 1 Search strategy in PubMed.**

| **Step** | **Query** | **Results** |
| --- | --- | --- |
| #1 | Continuous passive motion | 705 |
| #2 | Physical therapy OR Active exercise OR active rehabilitation | 596904 |
| #3 | Clinical trial OR Clinical study | 967243 |
| #4 | #1 AND #2 AND #3 AND | 447 |

**Query:**

"motion therapy, continuous passive"[MeSH Terms] AND ((("activable"[All Fields] OR "activate"[All Fields] OR "activated"[All Fields] OR "activates"[All Fields] OR "activating"[All Fields] OR "activation"[All Fields] OR "activations"[All Fields] OR "activator"[All Fields] OR "activator s"[All Fields] OR "activators"[All Fields] OR "active"[All Fields] OR "actived"[All Fields] OR "actively"[All Fields] OR "actives"[All Fields] OR "activities"[All Fields] OR "activity s"[All Fields] OR "activitys"[All Fields] OR "motor activity"[MeSH Terms] OR ("motor"[All Fields] AND "activity"[All Fields]) OR "motor activity"[All Fields] OR "activity"[All Fields]) AND ("exercise"[MeSH Terms] OR "exercise"[All Fields] OR "exercises"[All Fields] OR "exercise therapy"[MeSH Terms] OR ("exercise"[All Fields] AND "therapy"[All Fields]) OR "exercise therapy"[All Fields] OR "exercise s"[All Fields] OR "exercised"[All Fields] OR "exerciser"[All Fields] OR "exercisers"[All Fields] OR "exercising"[All Fields])) OR (("activable"[All Fields] OR "activate"[All Fields] OR "activated"[All Fields] OR "activates"[All Fields] OR "activating"[All Fields] OR "activation"[All Fields] OR "activations"[All Fields] OR "activator"[All Fields] OR "activator s"[All Fields] OR "activators"[All Fields] OR "active"[All Fields] OR "actively"[All Fields] OR "actives"[All Fields] OR "activities"[All Fields] OR "activity s"[All Fields] OR "motor activity"[MeSH Terms] OR ("motor"[All Fields] AND "activity"[All Fields]) OR "motor activity"[All Fields] OR "activity"[All Fields]) AND ("rehabilitant"[All Fields] OR "rehabilitants"[All Fields] OR "rehabilitate"[All Fields] OR "rehabilitated"[All Fields] OR "rehabilitates"[All Fields] OR "rehabilitating"[All Fields] OR "rehabilitation"[MeSH Terms] OR "rehabilitation"[All Fields] OR "rehabilitations"[All Fields] OR "rehabilitative"[All Fields] OR "rehabilitation"[MeSH Subheading] OR "rehabilitation s"[All Fields] OR "rehabilitator"[All Fields] OR "rehabilitators"[All Fields])) OR "Physical Therapy Modalities"[MeSH Terms]) AND "Clinical Trial"[Publication Type]
